# Supplementary material for: Effect of freeze-thaw cycles on mechanical performance of loess soil stabilized with nano magnesium oxide
Source: PLoS One. 2025 Apr 29;20(4):e0319909. doi: 10.1371/journal.pone.0319909 (PMC12040123; doi:10.1371/journal.pone.0319909)
Supplement: Attachment — Submitted filename: Response to Reviewers-2.doc [file pone.0319909.s004.doc]

**Answer/response to Reviewers Comments**

Dear Reviewer

Thank you for your comments on our manuscript entitled “Effects of freeze-thaw cycles on mechanical performance of loess soil stabilized with nano magnesium oxide” (ID: **PONE-D-24-44491**). Those comments are very helpful for revising and improving our paper, as well as the important guiding significance to other researches. We have studied the comments carefully and substantially revised our manuscript which we hope meet with approval. The main corrections are in the manuscript and the responds to the reviewers’ comments are as follows.

**Reviewer 1:**

**Comments to the Author**

In this study, the variation of mechanical properties of nano-MgO treated loess under freeze-thaw cycles was investigated through a series of unconfined compression tests. The topic of this research is interesting. However, the depth and novelty of this study needs to be further clarified. In addition, there are a number of grammatical and expression errors in the manuscript. The following issues are suggested to be revised before recommending publication.

1. The introduction does not highlight the innovation and necessity of this work. Many studies have investigated the effect of nano-MgO content and water content on the unconfined compressive strength of nano-MgO treated loess under freeze-thaw cycles.

2. In the unconfined compression tests, two samples were produced at each test condition. How to handle failure strength and strain data from two samples? The stress-strain curves of the samples were not present in the manuscript.

3. The description of the compaction test does not need to be included in Table 3.

4. In terms of mechanical properties and microstructure analysis, some relevant references may be referred, e.g., “Micro-scale investigations on the mechanical properties of expansive soil subjected to freeze-thaw cycles”, “Investigation of coupled thermo-hydro-mechanical processes on soil slopes in seasonally frozen regions”.

5. What is the use of prediction equations (7) and (8)?

6. In NMR analysis, what is non-freezable water and freezable water? Bound water and free water are used in many studies.

7. The freeze-thaw cycle can cause changes in the physical and mechanical properties of the rock and soil mass, the following literature might be referred. (2024). Seismic Response and Dynamic Failure Mode of a Class of Bedding Rock Slope Subjected to Freeze–Thaw Cycles. Rock Mechanics and Rock Engineering, 1-19.

1. The introduction does not highlight the innovation and necessity of this work. Many studies have investigated the effect of nano-MgO content and water content on the unconfined compressive strength of nano-MgO treated loess under freeze-thaw cycles.

**Response:**

**a. In responds to** “The introduction does not highlight the innovation and necessity of this work.”

Thank you for your kind suggestion to improve our manuscript. We have revised the introduction to highlight the innovation and necessity of this work.

The necessity of this work is illustrated in the first two paragraph. The first paragraph makes out a clear illustration of the point that it is imperative to develop low-carbon-footprints additives for loess stabilization. Nanoscale MgO (NM) is a promising alternative for construction engineers and designers

**b. In responds to** “Many studies have investigated the effect of nano-MgO content and water content on the unconfined compressive strength of nano-MgO treated loess under freeze-thaw cycles.”

Thank you very much for your valuable comments. We tracked and searched the correlative references about nano MgO, and loess, and freeze-thaw, with the first page most relevant references presented below. To the best of our knowledge, there is limited studies on the soil stabilization techniques based on nanomaterials, and it is the first time to investigate the effects of freeze-thaw cycles on mechanical performance of loess soil stabilized with nano magnesium oxide. Specifically, we have added the relevant reference, which help to further strengthen the background information and provide a more comprehensive context for our study.

2. In the unconfined compression tests, two samples were produced at each test condition. How to handle failure strength and strain data from two samples? The stress-strain curves of the samples were not present in the manuscript.

**Response:**

Thank you for your question.

For UCT, two samples were fabricated at each test condition to ensure consistency. To minimize the discreteness of test results, the difference between the actual and target dry density was controlled within 0.01 g/cm^3^, and the difference in water content was within 0.1%.

The compression tests were carried out under constant axial displacement rate of 0.8 mm/min, and continued until sample failure or 10% of axial strain achieved. The value of UCS is determined from either the maximum load attained per unit area or the load per unit area at 10% axial strain, whichever occurs first in the compression. If the discrepancy between the UCS of multiple tests exceeded 20% of the minimum value, the test was repeated to ensure accuracy and consistency.

For the two samples at same condition, the UCS, failure strain, and deformation modulus were determined separately. And the average value of these parameters was taken for further analysis, and the discrepancy was represented by the error bar in the bar graphs, such as Figs. 3, 4, and 5. For the clarity of our presentation, the stress-strain curves of the samples are represented by their key parameters, i.e. UCS, failure strain, and deformation modulus, we have discussed in the manuscript. Thank you again for your valuable feedback.

3. The description of the compaction test does not need to be included in Table 3.

**Response:**

Thank you for your kind suggestion. The compaction test in Table 3 was removed according to the reviewer’s suggestion.

4. In terms of mechanical properties and microstructure analysis, some relevant references may be referred, e.g., “Micro-scale investigations on the mechanical properties of expansive soil subjected to freeze-thaw cycles”, “Investigation of coupled thermo-hydro-mechanical processes on soil slopes in seasonally frozen regions”.

**Response:**

Thanks for your kind suggestions. We apologize for neglecting the relevant and important studies. We have added the relevant references as you suggested in the “Results and discussion” section (Line 193 and Line 353) to support our manuscript.

[35] Chen Q, Ghimire B, Su L, Liu, Y. Micro-scale investigations on the mechanical properties of expansive soil subjected to freeze-thaw cycles. Cold Regions Science and Technology, 2024; 219: 104128. <https://doi.org/10.1016/j.coldregions.2024.104128>

[42] Chen Q, Liu Y, Wang Y, Su L, Cheng, Y. Investigation of coupled thermo-hydro-mechanical processes on soil slopes in seasonally frozen regions. Cold Regions Science and Technology, 2025; 229: 104356. <https://doi.org/10.1016/j.coldregions.2024.104356>

5. What is the use of prediction equations (7) and (8)?

**Response:**

According to your suggestion, we have added some explanation for the proposed empirical expressions,

Line 322-333: “Taking the equations in Fig. 9 into Eq. (6), comprehensive functions considering three governing factors, $C_{\text{NM}}$, $w$, and *N*, are established as follows,

Model 1 is established for predicting the strength of NM-treated loess at a fixed *w* of 20%, the optimum water content of loess soil,

$\begin{matrix} q_{\text{u}}=519.6\cdot\left[1+\left(-5.401\cdot C_{\text{NM}}+0.518\right)\left[e^{\left(5.440\cdot C_{\text{NM}}-0.559\right)\cdot N}-1\right]\right] \\ \cdot\left[-853.55\cdot\left(C_{\text{NM}}^{2}-0.02^{2}\right)+39.23\cdot\left(C_{\text{NM}}-0.02\right)+1 \text{ }\right] \end{matrix}$ (7)

According to the formula, the unconfined compressive strength of stabilized loess with different NM content and FT cycles can be calculated during construction design.

Model 2. is established for evaluate the freeze-thaw resistance of NM-treated loess under the optimum NM dosage, i.e. $C_{\text{NM}}$ of 2%,

$\begin{matrix} q_{\text{u}}=519.6\cdot\left[1+\left(2.193\cdot w-0.026\right)\left[e^{\left(-2.103\cdot w-0.045\right)\cdot N}-1\right]\right] \\ \cdot\left[-21.90\cdot\left(w^{2}-0.2^{2}\right)+4.13\cdot\left(w-0.2\right)+1\right] \end{matrix}$ (8)

Based on the formula, the effects of freeze-thaw cycles on the unconfined compressive strength of stabilized loess with different water contents can be estimated. ”

6. In NMR analysis, what is non-freezable water and freezable water? Bound water and free water are used in many studies.

**Response:**

Thank you for your reminder. We have substituted the non-freezable water with bound water, and the freezable water with free water.

7. The freeze-thaw cycle can cause changes in the physical and mechanical properties of the rock and soil mass, the following literature might be referred.
(2024). Seismic Response and Dynamic Failure Mode of a Class of Bedding Rock Slope Subjected to Freeze–Thaw Cycles. Rock Mechanics and Rock Engineering, 1-19.

**Response:**

Thanks for your kind suggestions. We have added the relevant references as you suggested in the “Results and discussion” section (353) to support our manuscript.

[41] Liu Y, Cheng P, Liu E, Hu J, Cheng Y, Su L. Seismic Response and Dynamic Failure Mode of a Class of Bedding Rock Slope Subjected to Freeze–Thaw Cycles. Rock Mechanics and Rock Engineering, 2024; 1-19. <https://doi.org/10.1007/s00603-024-04318-2>

We believe that these revisions have improved the quality of our manuscript and addressed the concerns you raised. Please let us know if you have any further comments or suggestions.

Thank you once again for your time and effort in reviewing our manuscript.

Best regards,

Shufeng Chen
